# The Anti-Inflammatory Potential of Tricyclic Antidepressants (TCAs): A Novel Therapeutic Approach to Atherosclerosis Pathophysiology

**DOI:** 10.3390/ph18020197

**Published:** 2025-01-31

**Authors:** Majid Eslami, Marzieh Monemi, Mohammad Ali Nazari, Mohammad Hossein Azami, Parand Shariat Rad, Valentyn Oksenych, Ramtin Naderian

**Affiliations:** 1 Cancer Research Center, Semnan University of Medical Sciences, Semnan 35147-99442, Iran; m.eslami@semums.ac.ir; 2 Department of Basic Science, Faculty of Pharmacy and Pharmaceutical Science, Tehran Medical Science, Islamic Azad University, Tehran 19395-1495, Iran; 3 Student Research Committee, Faculty of Medicine, Iran University of Medical Sciences, Tehran 14496-1453, Iran; 4 Student Research Committee, Kermanshah University of Medical Sciences, Kermanshah 67158-47141, Iran; 5 Faculty of Medicine, University of Bergen, 5020 Bergen, Norway; 6 Clinical Research Development Unit, Kowsar Educational, Research and Therapeutic Hospital, Semnan University of Medical Sciences, Semnan 35147-99442, Iran

**Keywords:** tricyclic antidepressants (TCAs), atherosclerosis, pathophysiology, inflammasome, cytokine

## Abstract

Atherosclerosis, a chronic inflammatory disease, is driven by complex molecular mechanisms involving inflammatory cytokines and immune pathways. According to recent research, tricyclic antidepressants (TCAs), which are typically prescribed to treat depressive disorders, have strong anti-inflammatory effects. TCAs, including imipramine and amitriptyline, alter inflammatory signaling cascades, which include lowering the levels pro-inflammatory cytokines like TNF-α, IL-1β, and IL-6 and inhibiting NF-κB activation. By inhibiting the NLRP3 inflammasome and suppressing pathways including JAK/STAT, MAPK, and PI3K, these effects are produced, improving endothelial function and reducing oxidative stress. The intricacy of TCAs’ anti-inflammatory actions has demonstrated by the existence of contradictory findings about how they alter IL-6 levels. The dependence of the heterogeneity of the reaction on the use of particular TCAs and experimental settings is shown by the fact that some studies show reduced IL-6 production, while others indicate increases or no changes. This review explores the multifaceted mechanisms through which TCAs modulate inflammatory pathways. TCAs inhibit NF-κB activation, reduce oxidative stress, and suppress the production of key inflammatory mediators, including IL-6 and TNF-α. They also regulate Toll-like receptor (TLR) signaling and NOD-, LRR-, and NLR family pyrin domain-containing protein 3 (NLRP3) inflammasome activation, reducing the release of IL-1β and IL-18, critical drivers of endothelial dysfunction and plaque instability. Given their capacity to target critical inflammatory molecules and pathways, TCAs provide great potential in the therapy of atherosclerosis, particularly for individuals with associated depression and cardiovascular risk factors. Nevertheless, further research is essential to clarify the precise molecular mechanisms, resolve inconsistencies in current findings, and establish the clinical applicability of TCAs as anti-inflammatory agents in atherosclerosis management.

## 1. Introduction

Atherosclerosis is a chronic inflammatory disease characterized by the accumulation of lipids, inflammatory cells, and fibrous elements within the arterial wall, leading to the formation of atherosclerotic plaques. This condition is a significant contributor to cardiovascular diseases, including coronary artery disease and ischemic heart disease [1]. Atherosclerosis is a leading cause of morbidity and mortality worldwide. It is estimated that ischemic heart disease affects millions globally, with significant prevalence in populations with high rates of traditional risk factors. Recent studies highlight inflammation as a critical factor in the pathogenesis of atherosclerosis. In particular, the nucleotide-binding oligomerization domain (NOD)-, LRR-, and NLR family pyrin domain-containing protein 3 (NLRP3) inflammasome and cytokines such as TNF-α and IL-1 have been identified as significant contributors to the inflammatory processes that drive atherosclerosis progression [2]. Pro-inflammatory cytokines (such TNF-α, IL-1β, and IL-6) are released by activated macrophages and other immune cells, which increase inflammation. These cytokines enhance the inflammatory response, resulting in more immune cell recruitment, which feeds a vicious cycle that promotes endothelial dysfunction and plaque development. In healthy tissue repair processes, inflammation is resolved through mechanisms involving anti-inflammatory cytokines and the apoptosis of inflammatory cells. However, in atherosclerosis, this resolution is often impaired, leading to chronic inflammation that sustains plaque development and vulnerability [3].

Tricyclic antidepressants (TCAs) are a class of medications that have been used for decades in the treatment of various mood disorders, particularly major depressive disorder (MDD). They are named for their chemical structure, which consists of three fused rings. Despite the development of newer antidepressants, TCAs remain an important option for many patients due to their efficacy and unique mechanisms of action. TCAs work by inhibiting the reuptake of neurotransmitters and antagonizing muscarinic receptors, alpha-adrenergic receptors (ɑ-1 and ɑ2), and histamine receptors (H1 and H2). These medications are particularly effective in treating depression in older adults, achieving a recovery rate of 75% compared to 51% with placebo [4]. TCAs, such as amitriptyline, work by inhibiting the reuptake of neurotransmitters like serotonin and norepinephrine, which can influence inflammatory processes. Research indicates that serotonin can modulate immune responses, suggesting that TCAs may indirectly affect inflammation through their impact on serotonin levels [5]. Research indicates that TCAs can inhibit the release of pro-inflammatory cytokines from immune cells. One study demonstrated that TCAs significantly reduced the production of IL-6, IL-1β, and TNF-α in human blood monocytes [6]. Also, recent findings suggest that TCAs may induce inflammation through mechanisms involving the NLRP3 inflammasome, a key player in inflammatory responses. By targeting this pathway, TCAs could potentially reduce inflammation-related damage [7]. Atherosclerosis is increasingly recognized as a chronic inflammatory disease, where both the innate and adaptive immune systems play pivotal roles in lesion development. The interplay between immune responses and lipid metabolism provides a unified framework that bridges the cholesterol and injury hypotheses. Immune cells, such as macrophages and T cells, not only respond to modified low-density lipoproteins (LDLs) but also perpetuate vascular injury through cytokine production and oxidative stress. This refined understanding of atherogenesis highlights the importance of integrating immunological mechanisms into therapeutic strategies aimed at reducing cardiovascular risk. Despite significant advancements in understanding the pathophysiology of atherosclerosis, the role of TCAs as anti-inflammatory agents remains inadequately explored, with conflicting findings regarding their effects on inflammatory pathways such as IL-6 and TNF-α. This review aims to address these gaps by providing a comprehensive analysis of the molecular mechanisms underlying TCAs’ anti-inflammatory properties, focusing on their potential application in atherosclerosis management. This review seeks to provide a clearer view of the therapeutic value of TCAs [8,9].

### 1.1. Strategy Search

To compile the literature for this narrative review, a comprehensive search was conducted across multiple electronic databases, including PubMed, Scopus, Web of Science, and Google Scholar. The literature search focused on studies published with no language restrictions applied. The search strategy was designed to capture relevant research articles, clinical studies, and review papers.

The following inclusion criteria were applied to the literature selection process:

- Studies that investigated the effects of tricyclic antidepressants (TCAs) on inflammatory pathways, particularly in the context of cardiovascular diseases, including atherosclerosis.
- Articles published in peer-reviewed journals.
- Studies involving human subjects or animal models.
- Research studies that provided primary data or detailed analyses of molecular mechanisms. Although some observational and small-scale human studies exist, robust RCTs that assess the therapeutic potential of TCAs in cardiovascular diseases remain scarce or unavailable.
- Exclusion criteria were as follows:
- Studies that focused on antidepressants other than TCAs.
- Articles not available in full text or lacking methodological details.
- Research not related to inflammation or cardiovascular disease.

The following keywords were used in the search process:

“Tricyclic antidepressants”, “Atherosclerosis”, “Inflammatory pathways”, “NF-κB activation”, “TLRs”, “Adiponectin”, “Cytokines”, and “Endothelial dysfunction”.

Priority was given to studies that provided mechanistic insights, particularly those related to molecular signaling pathways, and clinical studies that evaluated the effects of TCAs on inflammation in cardiovascular contexts. The search was further refined to focus on articles that presented findings on the interaction between TCAs and specific inflammatory mediators, including TNF-α, IL-6, and NF-κB, as well as studies exploring the role of TLR signaling in the modulation of inflammation.

### 1.2. Limitations of the Study

The limitations of this study predominantly arise from the reliance on preclinical and animal model findings, which, while valuable for elucidating molecular mechanisms, lack direct clinical validation. The absence of robust randomized controlled trials assessing the anti-inflammatory effects of TCAs in cardiovascular diseases, particularly atherosclerosis, limits the translational applicability of the findings. Furthermore, the heterogeneity in experimental design across the reviewed studies, including differences in animal models, dosages, treatment durations, and endpoints, contributes to variability in the reported outcomes. This inconsistency is made particularly evident by the presence of conflicting data regarding the modulation of cytokines such as IL-6 and TNF-α by TCAs, underscoring the need for standardized methodologies in future research. The influence of confounding factors, such as genetic predispositions, comorbid conditions like depression and metabolic syndrome, and other individual variations, further complicates the interpretation of the results. Another significant limitation is the lack of long-term studies evaluating the safety, efficacy, and potential adverse effects of TCAs in cardiovascular contexts. Finally, the absence of detailed clinical evidence on how TCAs interact with existing anti-inflammatory or lipid-lowering therapies leaves an important gap in understanding their comprehensive therapeutic potential.

## 2. Mechanisms of Action

### 2.1. Reuptake Inhibition

TCAs block the serotonin transporter (SERT) and norepinephrine transporter (NET), preventing the reabsorption of these neurotransmitters back into presynaptic neurons. This leads to increased levels of serotonin and norepinephrine in the synaptic cleft, which is associated with improved mood and reduced symptoms of depression. Additionally, the delayed start of TCAs’ clinical effects indicates that downstream modifications such as receptor sensitivity adjustments, intracellular signaling modifications, and gene expression changes are also involved in their therapeutic advantages. The restoration of brain homeostasis and increased neuroplasticity, which are essential for attaining long-lasting antidepressant benefits, are believed to be facilitated by these long-term alterations. However, TCAs’ wide range of pharmacological characteristics, such as their affinity for adrenergic, muscarinic, and histaminergic receptors, adds to their adverse impact profile and makes their application in clinical practice extremely cautious [10].

### 2.2. Pharmacological Effects

Beyond reuptake inhibition, TCAs have been shown to interact with various receptors, including histamine, acetylcholine, and adrenergic receptors. These interactions can contribute to both therapeutic effects and side effects, such as sedation and anticholinergic effects. The anticholinergic effects of TCAs, such as dry mouth, constipation, urine retention, impaired vision, and cognitive impairment, are caused by the inhibition of muscarinic acetylcholine receptors. These side effects may worsen pre-existing diseases or cause disorientation and delirium, which can be especially problematic for older people. TCAs also block α1-adrenergic receptors, which causes vasodilation and orthostatic hypotension. This raises the risk of falls and dizziness, particularly in elderly people. This characteristic also adds to TCAs’ cardiovascular adverse effects, such as possible arrhythmias, which should be avoided in individuals who already have cardiac issues [11].

### 2.3. Modulation of Pain Pathways

TCAs are also effective in treating chronic pain conditions, suggesting that they may modulate pain pathways through mechanisms independent of their antidepressant effects. For instance, they may activate descending serotonergic pathways that influence endogenous opioid systems, providing analgesic benefits. One proposed mechanism involves the inhibition of serotonin and norepinephrine reuptake, which enhances descending pain-inhibitory pathways originating in the brainstem. TCAs enhance the endogenous inhibition of nociceptive signals by increasing the availability of these neurotransmitters in the spinal cord, which lessens pain perception. In diseases like those involving neuropathic pain, where disruption in these pathways is frequently seen, this activity is especially pertinent. TCAs interact with ion channels and receptors that affect pain signals in addition to inhibiting reuptake. For example, TCAs have been demonstrated to inhibit peripheral nerve sodium channels, which may decrease pain fiber hyperexcitability. Additionally, though these processes are less well understood, their regulation of the glutamatergic and γ-Aminobutyric acid (GABA)-ergic systems and antagonism of N-methyl-D-aspartate (NMDA) receptors may possibly contribute to their analgesic effects.

### 2.4. Impact on Neurotransmitter Systems

Studies indicate that TCAs may also affect other neurotransmitter systems beyond serotonin and norepinephrine, including having a modulating effect on potassium channels and antagonizing NMDA receptors. These actions could enhance their efficacy in treating conditions like fibromyalgia. The modulation of potassium channels is one significant strategy that could decrease hyperalgesia and normalize neural excitability. TCAs can reduce the excessive firing of pain-related neurons, which is a defining feature of diseases like fibromyalgia and neuropathic pain, by altering potassium channel activity. Patients with heightened central sensitization, where abnormal neural activity intensifies pain perception, may find this feature especially pertinent. TCAs also show antagonism to NMDA receptors, which is believed to contribute to their analgesic effects. One important mechanism of central sensitization and hyperalgesia in chronic pain conditions is the NMDA receptor. TCAs may prevent the brain and spinal cord from pathologically amplifying pain signals by inhibiting these receptors. Their ability to improve descending inhibitory pathways might function in association with this activity, making them even more useful in treating fibromyalgia and other pain conditions [12,13].

## 3. Inflammatory Pathways

Inflammatory and immune response pathways are critical biological processes that help the body respond to infections, injuries, and various diseases. These pathways involve complex interactions between cells, cytokines, and signaling molecules, which can either promote healing or contribute to chronic inflammatory conditions [14]. Inflammation can be triggered by various factors, including infections, tissue damage, or events like cardiac infarction. These causes may be either infectious or non-infectious in nature. When tissue injury occurs, the body initiates a series of chemical signals designed to promote the healing process. These signals draw leukocytes from the bloodstream to the site of injury, where they become activated and release cytokines, further driving the inflammatory response [15]. Cold exposure has been shown to downregulate immune response pathways in perivascular adipose tissue, which may influence vascular health. The inflammatory state of this tissue is linked to conditions like atherosclerosis. Astrocytes play a pivotal role in modulating immune responses within the central nervous system. The activation of TLRs on astrocytes can lead to pro-inflammatory responses via NF-κB signaling, highlighting the importance of these pathways in neuroinflammatory conditions [16]. Regarding the practical use of TCAs as anti-inflammatory agents, several clinical scenarios are proposed to highlight contexts where their therapeutic potential could be evaluated:

1. Patients with atherosclerosis and comorbid depression: Atherosclerosis, a condition marked by chronic inflammation, sees cytokines such as IL-6 and TNF-α playing critical roles in disease progression. TCAs, which influence these cytokines and reduce NF-κB activity, could be assessed as adjunctive treatments for patients suffering from both atherosclerosis and depression. Clinical trials in this setting could measure inflammatory biomarkers, endothelial function, and cardiovascular events, evaluating the combined effects of mood improvement and inflammation reduction.
2. Chronic inflammatory diseases with psychiatric comorbidities: Diseases like rheumatoid arthritis, inflammatory bowel disease, and psoriasis are characterized by systemic inflammation, which contributes to disease severity. These conditions are frequently accompanied by higher rates of depression and anxiety, which in turn exacerbate inflammatory responses. TCAs could be explored in these populations to assess their ability to reduce inflammation while simultaneously improving mental health. Clinical endpoints could include cytokine profiles, disease activity scores, and quality of life measures.
3. Post-acute coronary syndrome recovery: Inflammation following acute coronary syndromes, such as myocardial infarction, significantly contributes to adverse cardiovascular outcomes. TCAs may be examined as part of secondary prevention strategies for patients with elevated inflammatory markers, especially those with coexisting depressive symptoms. This clinical setting could focus on the impact of TCAs on inflammatory resolution, recurrence of cardiac events, and psychological well-being.
4. Chronic pain syndromes with inflammatory components: Conditions such as fibromyalgia and neuropathic pain often involve low-grade inflammation and increased cytokine activity. Given the established role of TCAs in managing chronic pain, clinical trials could investigate whether their anti-inflammatory effects improve their efficacy in reducing pain severity and enhancing functionality.
5. Cancer-related fatigue and inflammation: Cancer-related fatigue, a multifaceted condition, is often linked to systemic inflammation and cytokine dysregulation. In oncology, TCAs could be evaluated for their dual ability to alleviate depressive symptoms while mitigating inflammation. This approach could offer potential benefits for patients’ quality of life and treatment tolerance.
6. Patients with metabolic syndrome or obesity: Metabolic syndrome is associated with chronic low-grade inflammation, which heightens the risk of cardiovascular disease. TCAs might be explored as adjunctive therapies in these patients, focusing on their potential to reduce inflammatory markers like IL-6 and improve endothelial function, alongside their effects on depressive symptoms [17,18,19,20].

### 3.1. IL-6 Pathways, Effects, and the Role of TCAs

IL-6 is a multifunctional cytokine that plays a complex role in the body, acting as both a pro-inflammatory and anti-inflammatory agent depending on the context. Elevated levels of IL-6 are associated with an increased risk of cardiovascular diseases, particularly due to its strong inflammatory properties, which can contribute to the development and progression of atherosclerosis [21]. IL-6 is often referred to as “the central cytokine” within the innate immune system due to its pivotal role in regulating various biological processes, including cell proliferation and differentiation [22]. One of the key pathways by which IL-6 exerts its pro-inflammatory effects involves promoting the expression of adhesion molecules on endothelial cells [23]. This action facilitates the recruitment of inflammatory cells to sites of vascular injury, enhancing the inflammatory response [23]. Additionally, IL-6 stimulates the production of acute-phase proteins in the liver, which are crucial markers and mediators of inflammation [23]. This process is integral to the body’s defense mechanisms, although it can also contribute to pathological conditions such as atherosclerosis. Beyond these, IL-6 plays several other significant roles as a pro-inflammatory agent, including in the inhibition of Treg differentiation, the promotion of neutrophil and macrophage chemotaxis, the induction of T-cell proliferation and cytotoxic T-cell differentiation, and the stimulation of vascular endothelial growth factor (VEGF) production, which is involved in angiogenesis and vascular permeability [24,25,26].

To understand how IL-6 can exert such a broad range of actions, it is essential to consider its signaling mechanisms. IL-6 exerts its effects through two primary signaling pathways: classical signaling and trans-signaling. In classical signaling, IL-6 binds to the membrane-bound IL-6 receptor (IL-6R) on immune cells. This receptor complex then associates with glycoprotein 130 (gp130), initiating intracellular signaling cascades such as the Janus kinase–signal transducer and activator of transcription (JAK-STAT) pathway. This leads to the phosphorylation of STAT3, which translocates to the nucleus and promotes the transcription of genes involved in inflammation and immune responses. This signaling pathway is crucial for targeted and regulated immune response within specific immune cells. Conversely, in trans-signaling, IL-6 binds to a soluble form of the IL-6 receptor (sIL-6R), which can then interact with gp130 on the surface of various cell types that do not express the membrane-bound IL-6R. This allows IL-6 to exert its effects on a wider range of cells and tissues, thus amplifying the inflammatory response beyond the immune cells. This broadens the scope of IL-6’s influence, making it a significant player in systemic inflammation [27,28].

TCAs have been a cornerstone in the treatment of depression for over 50 years. Recently, they have also been found to exhibit specific anti-inflammatory effects, a discovery that has expanded their potential therapeutic uses beyond mood disorders. The anti-inflammatory effects of TCAs are now understood to be mediated through several mechanisms, including the inhibition of nuclear factor kappa B (NF-κB) activation. NF-κB is a key transcription factor involved in the production of various inflammatory cytokines. By inhibiting NF-κB, TCAs can reduce the production of these cytokines, thereby attenuating inflammation. Additionally, TCAs have been shown to reduce oxidative stress and improve endothelial function, contributing to their anti-inflammatory properties. These effects are observed in various parts of the body, from the central nervous system to peripheral blood vessels [29].

One of the areas where the anti-inflammatory effects of TCAs are particularly intriguing, yet controversial, is their influence on the IL-6 pathway and its implications for atherosclerosis. Research is ongoing to determine whether TCAs inhibit or stimulate the production of IL-6. Some studies suggest an inhibitory effect. For example, Cattaneo et al. [30] reported that treatment with nortriptyline significantly decreased IL-6 levels. Similarly, studies by Xia et al. and Duda et al. demonstrated that imipramine treatment resulted in reduced IL-6 levels. Conversely, other studies have presented conflicting evidence. Research by Kubera et al. [31], Fornaro et al. [32], and Lanquillon et al. indicated that treatment with TCAs such as imipramine, duloxetine, and amitriptyline led to increased IL-6 levels. Moreover, a study by Curzytek et al. [33] found that treatment with imipramine did not significantly alter IL-6 levels, highlighting the complexity and variability of the response.

Interpreting these data involves several signaling pathways, which are not yet fully understood but can be referred to through various mechanisms. One potential pathway is the suppression of NF-κB activation by TCAs. NF-κB is a crucial transcription factor involved in the inflammatory response. TCAs can also inhibit JAK and signal transducer and activator of transcription (STAT) proteins, which facilitate the translocation of NF-κB to the nucleus, where it promotes the expression of inflammatory genes. By inhibiting the JAK/STAT pathway, TCAs can reduce the activation of these inflammatory genes, subsequently decreasing the production of IL-6. Additionally, TCAs can inhibit the activation of the mitogen-activated protein kinase (MAPK) and phosphoinositide 3-kinase (PI3K) pathways, which are also implicated in the production of IL-6, thereby exerting an anti-inflammatory effect [6].

**Figure 1:**
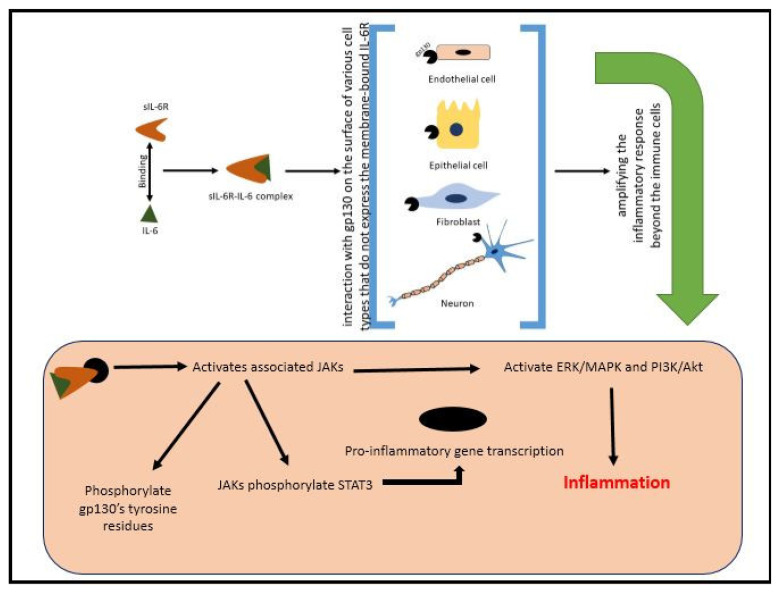
A figure illustrating the IL-6 trans-signaling pathway and clarifying its role in chronic inflammation.

In conclusion, there are currently insufficient data to definitively clarify the effect of TCAs on the progression of atherosclerosis through modulation of the IL-6 pathway. Future research is needed to determine whether TCAs can effectively decrease the production of IL-6. Should future studies confirm this hypothesis, it could be concluded that TCAs help to reduce inflammation and improve vascular health, making them a promising therapeutic option for patients with both depression and atherosclerosis, potentially improving patient outcomes. Until such data are available, the precise impact of TCAs on atherosclerosis remains unclear, and this represents a significant gap in our current understanding that needs to be addressed in future research endeavors (Figure 1).

Previous studies on the impact of TCAs on IL-6 production have reported inconsistent findings, highlighting the complexity of their mechanisms of action. Some studies have demonstrated that TCAs such as imipramine and nortriptyline significantly reduce IL-6 levels, likely through the inhibition of NF-κB activation and the modulation of the JAK/STAT and MAPK pathways. These mechanisms may lead to decreased transcription and release of IL-6 in inflammatory settings. Conversely, other studies report increased or unchanged IL-6 levels following TCA treatment. These discrepancies may arise from differences in experimental conditions, such as variations in the cell types, animal models, or patient populations studied. For instance, the pro-inflammatory or anti-inflammatory roles of IL-6 depend on the balance between its classical and trans-signaling pathways, which may be differentially affected by TCAs. Furthermore, the dose and duration of TCA treatment could influence the extent of IL-6 modulation. Higher doses or prolonged exposure may activate compensatory mechanisms, such as the upregulation of soluble IL-6 receptors that amplify IL-6 activity through trans-signaling. Additionally, the interplay of TCAs with underlying comorbidities, such as depression or metabolic disorders, may alter cytokine profiles, contributing to the variability observed across studies.

**Figure 2:**
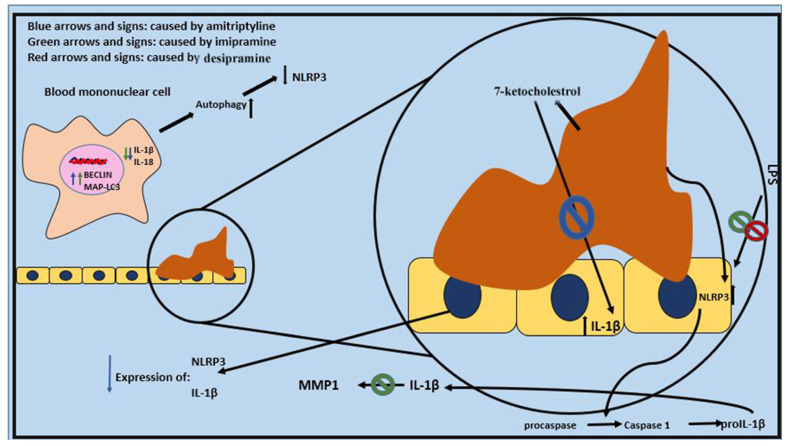
Amitriptyline and imipramine can induce autophagy, which itself can lead to a decrease in NLRP3 activation. The figure there is an illustration of endothelium, a plaque of cholesterol, their related pathways, and the reactions that TCAs can affect. Lipopolysaccharide (LPS) is another molecule that induces the production of NLRP3 and its effect can be inhibited by some TCAs.

**Figure 3:**
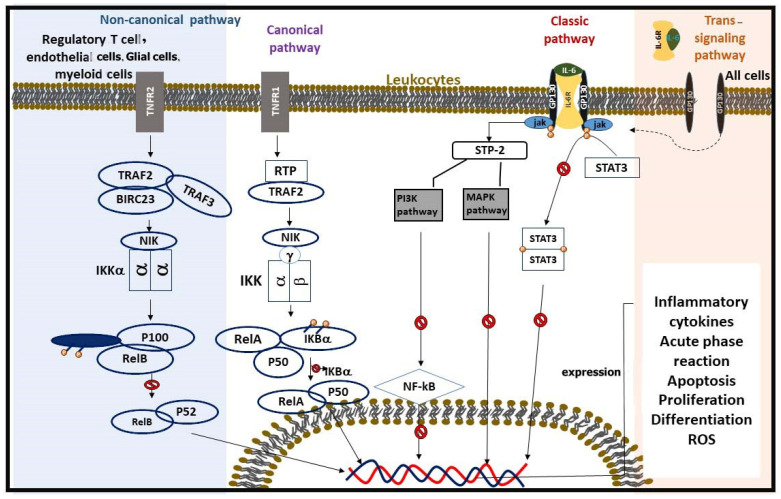
This figure demonstrates the trans-signaling and classic pathways of IL-6 signaling along with the canonical and non-canonical NF-κB pathways in different cells. The interaction of TCA drugs at various points in these pathways is also depicted, showing the blockade of signaling processes that lead to inflammatory responses that can lead to atherosclerosis.

Resolving these contradictions requires standardized methodologies and a deeper exploration of the molecular pathways involved. Future research should also focus on stratifying results by patient characteristics and treatment regimens to identify the context-dependent effects of TCAs on IL-6 production [6], (Figure 2 and Figure 3) (Table 1). 

### 3.2. TNF-α in Atherosclerosis and the Modulatory Role of TCAs

TNF-α is one of the most critical cytokines involved in inflammation, produced by various cell types including macrophages, lymphocytes, and endothelial cells. It plays a central role in immune responses and various cellular processes such as cell proliferation, differentiation, and apoptosis. The involvement of TNF-α in atherosclerosis can be described through multiple mechanisms. For instance, TNF-α upregulates the expression of vascular cell adhesion molecule-1 (VCAM-1), intercellular adhesion molecule-1 (ICAM-1), and E-selectin on endothelial cells. This upregulation facilitates the adhesion and transmigration of leukocytes into the sub-endothelial space, a key step in the formation of atherosclerotic plaques. Additionally, TNF-α promotes the differentiation of monocytes into macrophages and subsequently into foam cells. Moreover, TNF-α induces the production of other pro-inflammatory cytokines such as IL-1β, IL-6, chemokines, and matrix metalloproteinases (MMPs), all of which contribute to the inflammatory milieu within atherosclerotic plaques [21,22].

Highlighting TNF-α’s relationship with NF-κB, an essential regulator of inflammation, is crucial to understanding the processes by which it affects atherosclerosis. The NF-κB pathway is activated when TNF-α interacts with its receptors, tumor necrosis factor receptor 1 (TNFR1) and TNFR2. This activation leads to the transcription of genes involved in inflammation, apoptosis, and cell survival. NF-κB activation can occur via two pathways: canonical and non-canonical. In the canonical pathway, the phosphorylation of the inhibitor of nuclear factor kappa B (IκBα) allows NF-κB to translocate to the nucleus and activate target genes. In contrast, the non-canonical pathway involves the processing of NF-κB2/p100 to p52, which then forms a complex with RelB and translocates to the nucleus to initiate gene transcription. Following the activation of NF-κB, oxidative stress and apoptosis further propagate inflammation. TNF-α enhances the production of reactive oxygen species (ROS) through the activation of NADPH oxidase. ROS not only cause direct damage to endothelial cells but also inactivate nitric oxide (NO), leading to endothelial dysfunction. Furthermore, TNF-α can induce apoptosis in various cell types, including endothelial cells and smooth muscle cells, thereby contributing to plaque instability. This apoptotic process is mediated through both extrinsic (death receptor) and intrinsic (mitochondrial) pathways [39].

The regulation of TNF-α-related pathways is one potential mechanism for the anti-inflammatory actions of TCAs, as was previously noted. Most research shows that TCAs lower TNF-α levels in the blood. For instance, Xia et al. [40] reported a twofold decrease in TNF-α levels following treatment with amitriptyline. Other studies, such as those conducted by Fornaro et al. [32], Cattaneo et al. [30], and Lin et al. [41], have corroborated these findings. TCAs inhibit NF-κB activation by preventing the degradation of IκBα, thus reducing the transcription of pro-inflammatory genes regulated by NF-κB, including TNF-α, IL-1β, IL-6, and other pro-inflammatory cytokines. Additionally, TCAs reduce oxidative stress by scavenging ROS, highlighting one of their antioxidant properties. They also enhance the activity of antioxidant enzymes such as superoxide dismutase (SOD) and catalase, further protecting endothelial cells from oxidative damage. Collectively, these actions help restore NO bioavailability and inhibit apoptosis, leading to improved endothelial function, enhanced vasodilation, and a decreased likelihood of atherosclerotic plaque formation [40,42].

Cytokines in the TNF family, such as TNF-α, play a pivotal role in regulating inflammation, cell survival, proliferation, and differentiation. These cytokines initiate rapid transcriptional responses by activating the NF-κB signaling pathway, a central regulator of inflammatory and immune responses. Upon binding to their receptors (e.g., TNFR1 and TNFR2), TNF-family cytokines activate downstream signaling cascades, leading to the phosphorylation and degradation of IκB proteins. This degradation releases NF-κB dimers, primarily composed of p65 and p50 subunits, allowing them to translocate to the nucleus [43,44]. Once in the nucleus, NF-κB promotes the transcription of target genes involved in various processes, including the production of pro-inflammatory cytokines (e.g., IL-6, IL-1β), adhesion molecules (e.g., ICAM-1, VCAM-1), and enzymes involved in oxidative stress and matrix remodeling. This rapid activation and gene regulation are critical for the body’s response to injury or infection. However, sustained activation of this pathway, as observed in chronic inflammatory diseases like atherosclerosis, can lead to tissue damage and plaque instability. In the context of this review, understanding the modulation of TNF-induced NF-κB activation by TCAs is essential. TCAs have been shown to inhibit NF-κB activation by stabilizing IκB proteins, reducing TNF-α production, and mitigating downstream inflammatory effects. This highlights their potential therapeutic role in conditions where excessive NF-κB activation contributes to disease progression, such as atherosclerosis (Table 2) [45].

### 3.3. NF-κB Pathway and the Effects of TCAs

NF-κB is an essential transcription factor intricately linked to inflammatory responses, such as those observed in atherosclerosis. NF-κB regulates the transcription of numerous genes that play specific roles in atherosclerosis, including cytokines, chemokines, adhesion molecules, acute phase proteins, and regulators of apoptosis and cell proliferation [47].

The NF-κB family encompasses several proteins, such as RELA (p65), NF-κB1 (p50; p105), NF-κB2 (p52; p100), c-REL, and RELB. These proteins possess a conserved amino-terminal 300-amino-acid region, which includes domains for dimerization, nuclear localization, and DNA binding. Activation of NF-κB proteins occurs through binding with IκB proteins, such as IκBα, IκBβ, and IκBε [48].

NF-κB activation can occur via two distinct pathways: the canonical (or classical) pathway, regulated by IκB degradation, and the non-canonical (or alternative) pathway, mediated by p100. In the canonical pathway, NF-κB dimers are bound to IκB proteins within the cytosolic compartment of the cell. This pathway is activated by stimuli such as interleukins, TNF-α, or LPS. Activation leads to the engagement of the IKK complex, which consists of IKKα, IKKβ, and IKKγ subunits. Current evidence suggests that IKKα and IKKβ play catalytic roles, while IKKγ (NEMO) has a regulatory function. A kinase cascade activates the IKK complex, resulting in the phosphorylation of IκB. The IKKβ subunit is primarily responsible for this phosphorylation process. Ultimately, the polyubiquitination and degradation of IκB by the proteasome lead to the release of NF-κB [49].

The non-canonical pathway is stimulated by factors such as CD40 ligation, lymphotoxin-β, and B-cell activating factor. This pathway manages the ubiquitin-mediated processing of p100 and regulates the levels of relB-p52 heterodimers. Unlike the canonical pathway dimers, relB-p52 dimers do not bind to IκB proteins but rather function in the cytoplasm as relB-p100 dimers. The processing of p100 to p52 in this pathway relies on signaling through IKK1 and NF-κB-inducing kinase (NIK), allowing relB-p52 dimers to translocate to the nucleus and regulate the transcription of NF-κB-dependent genes. Upon activation, NF-κB translocates to the nucleus, initiating the transcription of target genes, including those for cell adhesion molecules and pro-inflammatory factors. NF-κB activation can be physiological or pathological. The presence of p65 nuclear translocation in atherosclerotic lesions, enhanced NF-κB activity in unstable coronary atherectomies, and colocalization with target gene expression illustrate the link between NF-κB activation and atherosclerosis [50,51].

**Figure 4:**
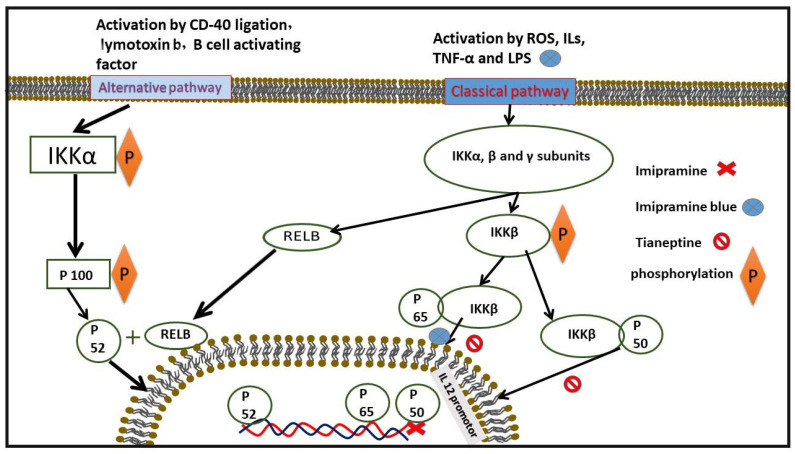
This figure illustrates the inhibitory effects of some TCAs on the NF-κB pathway and exactly where they affect, at both protein and gene expression levels.

NF-κB has a significant relationship with tricyclic antidepressant drugs. TCAs are known to decrease NF-κB activation. One mechanism through which TCAs reduce NF-κB activity is by inhibiting the release of TNF-α [29]. Studies have shown a decrease in total NF-κB and the nuclear/total NF-κB ratio found via western blot analysis as a result of imipramine treatment. Additionally, remodeling the IL-10 promoter to inhibit p50/c-Rel binding is another mechanism by which imipramine exerts its effects. Imipramine blue (IB) reduces NADPH oxidase activity, leading to decreased ROS production [52]. ROS are crucial activators of NF-κB. IB treatment also inhibits the nuclear translocation of p65 in HNSCC cell lines, OECM-1, and SAS [52]. Another TCA, tianeptine, reduces the activation of p50, p65, and IκB, all of which play essential roles in NF-κB activation. Moreover, amitriptyline treatment has been shown to suppress NF-κB activity in rats [53], (Figure 4, Table 3).

### 3.4. Toll-like Receptors (TLRs) in Atherosclerosis and Modulation by TCAs

TLRs are a class of pattern recognition receptors that play a crucial role in inflammation and the innate immune response. The expression of TLRs has been associated with atherosclerosis (AS) and other cardiovascular diseases. TLRs become activated by recognizing pathogen-associated molecular patterns (PAMPs) and damage-associated molecular patterns (DAMPs).

The signaling pathways mediated by TLRs are complex, and their mechanisms are not fully understood. TLRs operate through two primary signaling pathways: the MyD88-dependent pathway (involving TLRs 1, 2, 4, 5, 6, 7, 8, and 9) and the TRIF-dependent pathway (involving TLRs 3 and 4). Typically, TLR activation initiates immune responses through the MyD88-dependent pathway. After TLR activation, the interaction between MyD88 and the TIR domain of TLRs forms a complex that subsequently activates Interleukin-1 receptor-associated kinase (IRAK). IRAK4, a kinase, enhances the phosphorylation of IRAK1 and recruits phosphorylated IRAK1 and TRAF6 [58].

An alternative pathway is the TRIF-dependent pathway. This pathway involves multiple proteins, including TRIF, TRAF6, receptor-interacting serine/threonine-protein kinase 1 (RIP1), receptor-associated death domain protein (TRADD), and TNF. TLR3 functions by binding to TRIF, while TLR4 functions by binding to TRIF through TRAM, regulated by this pathway. TRIF activates TANK-binding kinase 1 (TBK1), which activates IRF3. Activated IRF3 is subsequently translocated into the nucleus, where it may act as a regulator of IFN-β synthesis. Additionally, the TRIF-dependent pathway activates the conversion of TRAF6 to the NF-κB pathway to trigger immune responses [59].

**Figure 5:**
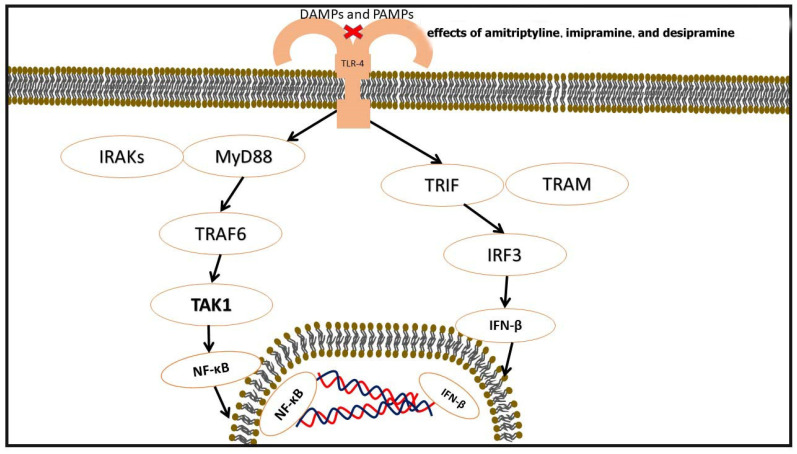
This figure is a brief view of some pathways related to TLR-4, a receptor that can be inhibited by amitriptyline, imipramine, and desipramine dose-dependently.

TLRs play a significant role in the onset and progression of atherosclerosis. TLR signaling pathways in atherosclerosis stimulate various cells with different signals. In endothelial cells, TLR activation increases the expression of NF-κB, ROS, IL-8, VCAM, IP-10, and P-selectin, contributing to the development of atherosclerosis. In platelets, TLR activation increases the expression of CD40L, CCL5, ERK1/2, PI3K/AKT, and NF-κB. Monocytes (increasing MAFB, ISGs, and CXCL-4), macrophages (elevating MMP2/9, CCR2, ICAM1, VCAM1, and MCP1), dendritic cells (increasing NF-κB, IL-6, IL-2, CD80, and CD86), and foam cells (elevating TN-C, ROS, the TLR4-Src pathway, and TREMs) are other cell types that TLRs stimulate to induce atherosclerosis. A study on diabetic and non-diabetic rats by Habib et al. demonstrated a decrease in aortic TLR-4 gene and protein expression in non-diabetic rats as a result of imipramine treatment. Another study showed the dose-dependent inhibition of TLR-4 and TLR-2 by amitriptyline, imipramine, and desipramine, in order of inhibitory potency [60,61], (Figure 5).

#### Signaling Pathways Mediated by TLRs

TLRs play a pivotal role in immune regulation and inflammatory responses, particularly in the pathogenesis of atherosclerosis. These receptors recognize PAMPs and DAMPs, triggering complex signaling cascades that amplify inflammatory responses. Two primary pathways mediate TLR signaling: the MyD88-dependent pathway and the TRIF-dependent pathway. The MyD88-dependent pathway is utilized by most TLRs, including TLR1, 2, 4, 5, 6, 7, 8, and 9. Upon activation, TLRs recruit the adaptor protein MyD88 through their TIR domains [62]. MyD88 subsequently recruits and activates IRAKs, particularly IRAK4 and IRAK1. This activation facilitates the interaction with TRAF6, leading to the downstream activation of NF-κB and MAPKs. The activation of NF-κB is critical as it promotes the transcription of pro-inflammatory genes, including cytokines such as TNF-α, IL-6, and IL-1β. In contrast, the TRIF-dependent pathway is predominantly associated with TLR3 and partially with TLR4. In this pathway, the adaptor protein TRIF interacts directly with TLR3 or indirectly with TLR4 through TRAM. TRIF activation leads to the stimulation of TANK-binding kinase 1 (TBK1) and interferon regulatory factor 3 (IRF3), driving the production of type I interferons, such as IFN-β. Additionally, TRIF activates TRAF6 and receptor-interacting serine/threonine-protein kinase 1 (RIP1), further stimulating the NF-κB pathway and amplifying immune responses [63].

The role of TLR signaling in atherosclerosis is significant. In endothelial cells, TLR activation enhances the expression of NF-κB, ROS, IL-8, VCAM, ICAM, and P-selectin, contributing to endothelial dysfunction and inflammation. In platelets, TLR signaling increases the expression of CD40L, CCL5, ERK1/2, PI3K/AKT, and NF-κB, promoting platelet aggregation and activation. Monocytes and macrophages, upon TLR stimulation, produce inflammatory mediators such as MAFB, ICAM1, VCAM1, and MCP-1, contributing to foam cell formation. Foam cells further exacerbate inflammation through increased production of TN-C, ROS, and TLR4-Src pathway activity. These processes collectively drive the progression of atherosclerosis [64].

TCAs, including imipramine, amitriptyline, and desipramine, have been shown to modulate TLR signaling, particularly through the inhibition of TLR2 and TLR4 expression in a dose-dependent manner. Imipramine has been observed to reduce TLR4 gene and protein expression in non-diabetic rats, while amitriptyline and desipramine exhibit inhibitory effects on TLR2 and TLR4, with amitriptyline demonstrating the high-est potency. This modulation of TLR signaling by TCAs reduces the activation of NF-κB, diminishes the production of pro-inflammatory cytokines, and alleviates oxi-dative stress and endothelial dysfunction. These anti-inflammatory effects highlight the potential of TCAs in managing inflammatory conditions, such as atherosclerosis. Through their ability to attenuate TLR-mediated inflammatory pathways, TCAs offer a promising therapeutic approach to mitigate vascular inflammation and improve cardiovascular health. The findings underscore the broader implications of TLR signaling and its modulation as a critical target for treating inflammation-driven diseases [60], (Table 4).

**Table 4:** TLRs in inflammation and atherosclerosis: modulation by TCAs.

Category	Details	Ref.
TLR Pathways	MyD88-Dependent Pathway: Involves TLRs 1, 2, 4, 5, 6, 7, 8, 9. Activates IRAK4, IRAK1, and TRAF6. TRIF-Dependent Pathway: Involves TLRs 3 and 4. Activates TBK1 and IRF3 for IFN-β synthesis. Converts TRAF6 to NF-κB.	[65]
Impact on Atherosclerosis	TLR activation in endothelial cells increases NF-κB, ROS, IL-8, VCAM, and P-selectin. In platelets, ↑ CD40L, ↑ CCL5, ↑ ERK1/2, and ↑ NF-κB. In monocytes, macrophages, and foam cells, it upregulates inflammatory cytokines and adhesion molecules.	[61]
Effects of TCAs on TLRs	Imipramine decreases TLR-4 gene and protein expression in non-diabetic rats. Amitriptyline, imipramine, and desipramine inhibit TLR-2 and TLR-4 in a dose-dependent manner. Order of potency: amitriptyline > imipramine > desipramine.	[66]
Therapeutic Implications	TCAs modulate TLR activity, reducing inflammatory responses. Potential application in treating inflammation-related cardiovascular diseases, such as atherosclerosis.	[61]

### 3.5. Adiponectin in Atherosclerosis and Effects of TCAs

Adiponectin, a hormone secreted by adipocytes, plays a crucial role in maintaining metabolic and cardiovascular health. It is considered a protective adipokine due to its potent anti-atherogenic effects, which are primarily mediated through its interaction with various signaling pathways. These pathways, including AMP-activated protein kinase (AMPK) and cAMP-dependent protein kinase (cAMP-PKA), are involved in regulating crucial cellular processes such as apoptosis, oxidative stress, and inflammation. By activating these pathways, adiponectin suppresses the production of ROS and the activation of the NF-κB pathway, both of which are key drivers of atherosclerosis. Additionally, adiponectin induces the production of NO, a molecule that helps maintain endothelial function and vasodilation, further enhancing its protective cardiovascular effects. TCAs, a class of medications commonly used to treat depression and anxiety disorders, have been shown to interact with various metabolic and cardiovascular systems. The effects of TCAs on adiponectin, however, remain an underexplored area of research. In particular, the influence of TCAs on adiponectin expression could have significant implications for patients with metabolic or cardiovascular diseases [67]. Several studies have addressed this topic, albeit with conflicting results. Doxepin, a TCA, has been shown to suppress adiponectin expression in a mouse model, suggesting a potential negative impact on adiponectin-related protective pathways. Doxepin is known to exert its effects through serotonin and norepinephrine reuptake inhibition, which could, in turn, affect metabolic pathways regulated by adiponectin. The suppression of adiponectin could potentially exacerbate the risk of atherosclerosis in patients using TCAs, particularly those already at risk due to preexisting metabolic conditions such as obesity or diabetes [68].

Imipramine, another TCA, has not significantly affected adiponectin expression in an in vitro setting. This suggests that the effects of TCAs on adiponectin may vary depending on the specific TCA used. The contrasting results between doxepin and imipramine may be explained by differences in their pharmacological profiles, dosage, and mechanisms of action. This highlights the complexity of understanding the full impact of TCAs on adiponectin, as different TCAs may have distinct effects on metabolic and cardiovascular health [69]. Recent research also underscores the importance of considering patient-specific factors when evaluating the effects of TCAs on adiponectin. These factors include genetic predisposition, preexisting metabolic conditions, and comorbidities that may modulate the expression and function of adiponectin. For example, patients with metabolic syndrome, a condition characterized by insulin resistance, obesity, and dyslipidemia, may be particularly vulnerable to the negative effects of TCAs on adiponectin levels. Conversely, patients without these conditions may experience a neutral or even beneficial impact on adiponectin expression. The clinical implications of these findings are significant. Given that adiponectin plays a protective role in cardiovascular health, TCAs that suppress adiponectin expression may exacerbate the risk of atherosclerosis in vulnerable patients. In contrast, TCAs with minimal or neutral effects on adiponectin could be safer options for individuals with underlying cardiovascular or metabolic conditions. Therefore, understanding the specific effects of different TCAs on adiponectin and its associated pathways is essential for optimizing antidepressant therapy, particularly in patients at high cardiovascular risk [70].

In conclusion, while initial studies suggest that TCAs may modulate adiponectin expression, further research is needed to fully elucidate the mechanisms underlying this interaction. Long-term clinical trials and studies examining the molecular mechanisms involved will be critical in determining the impact of TCAs on adiponectin and its subsequent effects on cardiovascular and metabolic health. Furthermore, personalized treatment approaches that consider both the mental health needs and cardiovascular risks of patients will be crucial in ensuring the safe and effective use of TCAs in clinical practice [71], (Table 5).

### 3.6. NLRP3 Inflammasome and the Effects of TCAs

The activation of the NLRP3 inflammasome by various distinct factors and cellular events initiates its assembly process. This activation converts pro-caspase-1 into its active form, caspase-1. Active caspase-1 then processes pro-(IL)-1β and pro-IL-18 into their active cytokine forms, IL-1β and IL-18. The release of these cytokines triggers a series of inflammatory responses, leading to endothelial dysfunction, increased oxidative stress, and the development of atherosclerosis. According to Karasawa et al. [75] and studies conducted by Duewell et al. [76], and Rajamäki et al. [77], one of the factors that induces activation of NLPR3 is the presence of cholesterol crystals.

In two related studies conducted by Alcocer-Gómez et al., it was proved that amitriptyline and imipramine can lower IL-1β, IL-18, the activation of the inflammasome, and the expression of NLRP3 mRNA in blood mononuclear cells of patients who have major depressive disorder. They can also enhance the expression of MAP-LC3 and BECLIN 1. These genes cause autophagy, which can in turn inhibit NLRP3 inflammasome (Table 6).

Bauer et al. [81] suggested that imipramine can prevent IL-1β from inducing the production of MMP-1, a molecule that can exacerbate atherosclerosis severity and lead to the rupture of atherosclerotic plaques. Cattaneo et al. indicated an association between a lack of response to nortriptyline and higher baseline mRNA levels of IL-1β, making this factor a potential predictor of treatment response. Similar results were achieved in their earlier study three years prior [30].

Chang et al. reported different results, noting elevated levels of IL-1 in obese mice treated with doxepin. In another study, Jiang et al. [82] induced acid sphingomyelinase activity with adenosine triphosphate (ATP) and lipopolysaccharide in J774A.1 cells and tested the inhibitory effect of imipramine on this pathway, which can lead to higher expression of NLRP3 and IL-1β. They found that imipramine could lower the expression of NLRP3 and IL-1β at both mRNA and protein levels. Koka et al. [83] had similar conclusions regarding the effect of amitriptyline in the carotid arterial endothelial cells of mice. Mello et al. [84] also demonstrated that imipramine could prevent the elevation of IL-1β after induction by lipopolysaccharide in mice, with a similar effect reported for desipramine by Obuchowicz et al. [85]. Sadeghi et al. [86] induced the production of IL-1β using carrageenan in rats and then used amitriptyline to measure its inhibitory effect on the production of this inflammatory molecule, describing the effect as significant. Sobieszczańska et al. [87] confirmed the inhibitory effect of clomipramine on the production of IL-1β in their study on mice. Yuan et al. [88] showed that amitriptyline can alleviate the increase in NLRP3 inflammasome formation, caspase-1 activity, and interleukin-1β levels due to 7-ketocholesterol. Table 7 summarizes the roles of critical molecules and pathways in the pathogenesis of atherosclerosis, the effects of TCAs on these targets, and the mechanisms through which TCAs exert their modulatory actions, highlighting their potential therapeutic applications beyond depression management.

## 4. Comprehensive Comparison of Contradictory Results in the Literature and Future Studies

The discovery of the contributions of the innate and adaptive immune systems has profoundly enhanced our understanding of atherogenesis, offering a conceptual bridge between the cholesterol and injury hypotheses. Inflammatory processes initiated by modified LDL uptake and the activation of pattern recognition receptors, such as TLRs, underscore the role of the innate immune system. Concurrently, adaptive immunity, exemplified by the activation of T cells and the production of pro-atherogenic cytokines like IFN-γ, contributes to lesion progression. This dual involvement explains why lipid-lowering therapies alone may be insufficient to halt atherosclerosis, emphasizing the need for anti-inflammatory interventions. TCAs, through their modulation of inflammatory pathways, could offer a novel therapeutic approach that aligns with this integrated model of atherogenesis. For example, their ability to inhibit NF-κB activation and reduce cytokine production may mitigate both innate and adaptive immune responses, addressing not only lipid-driven inflammation but also vascular injury. Further exploration of these mechanisms in the context of immune-mediated atherogenesis could provide a comprehensive framework for understanding TCAs’ potential in cardiovascular disease management [89].

The contrasting findings on the effects of TCAs on key inflammatory parameters, such as IL-6 and TNF-α levels, highlight the complexity of their mechanisms and the influence of varying experimental conditions. Differences in study designs, including the use of distinct TCA types, dosages, and treatment durations, may significantly contribute to these inconsistencies. For example, studies on imipramine and amitriptyline have demonstrated reductions in IL-6 and TNF-α levels, yet some investigations have reported either increased levels or no significant changes. These variations may be attributed to differences in cell lines, animal models, or patient populations, each of which exhibits unique inflammatory profiles [90].

Furthermore, the molecular pathways targeted by TCAs likely vary across experimental conditions. The modulation of IL-6 levels, for instance, could depend on the balance between classical and trans-signaling pathways. TCAs might preferentially inhibit specific pathways under certain conditions, leading to divergent outcomes. Similarly, variability in NF-κB pathway activation or oxidative stress levels may further complicate the interpretation of results [91].

Another possible explanation lies in the interplay between TCAs and comorbid conditions such as depression and metabolic disorders, which can independently alter inflammatory markers. Additionally, the influence of genetic factors, such as polymorphisms in cytokine or receptor genes, may account for interindividual variability in responses to TCAs. To resolve these contradictions, future research should adopt standardized protocols and integrate advanced techniques, such as single-cell transcriptomics or proteomics, to elucidate context-dependent mechanisms. Moreover, stratified analyses based on demographic and clinical characteristics could provide deeper insights into the variability of TCA effects, paving the way for personalized therapeutic strategies [92].

Future studies should aim to address the existing gaps in knowledge regarding the anti-inflammatory role of tricyclic antidepressants (TCAs) and their therapeutic potential in the management of atherosclerosis. Standardizing experimental conditions, including consistent dosages, treatment durations, and drug types, is essential to resolving discrepancies in findings related to the modulation of cytokines such as IL-6 and TNF-α. Furthermore, detailed mechanistic studies are necessary to explore the molecular pathways targeted by TCAs, particularly the NF-κB, JAK/STAT, and inflammasome activation pathways. Advanced techniques, such as single-cell RNA sequencing and proteomics, could provide more-precise insights into cell-specific effects. Furthermore, future studies should investigate the impact of comorbid conditions, such as depression, diabetes, and metabolic syndrome, on the anti-inflammatory actions of TCAs in atherosclerosis. This approach would help clarify the interplay between the psychiatric and cardiovascular benefits of these drugs. Special attention should also be given to the classical and trans-signaling pathways of IL-6 to determine how TCAs differentially regulate these mechanisms under various conditions [93].

Moreover, clinical trials should stratify participants based on demographic, genetic, and clinical characteristics to identify subpopulations most likely to benefit from TCA therapy. Long-term studies are also needed to evaluate the safety and efficacy of TCAs in cardiovascular diseases, with a specific focus on their impact on atherosclerosis progression, plaque stability, and potential adverse effects. Exploring the synergistic effects of TCAs with other anti-inflammatory or lipid-lowering treatments, such as statins, may uncover novel strategies for managing atherosclerosis. Additionally, identifying biomarkers, such as baseline cytokine levels or genetic polymorphisms, to predict treatment responses could facilitate personalized therapeutic approaches. Finally, translational research and large-scale randomized controlled trials are required to evaluate the clinical efficacy of TCAs in atherosclerosis, focusing on outcomes such as inflammation reduction, plaque stabilization, and cardiovascular events [94].

## 5. Conclusions

Chronic inflammation is a key component of the pathogenesis of atherosclerosis, which continues to be a major cause of morbidity and death globally. Plaque instability, immune cell recruitment, and endothelial dysfunction are all influenced by pro-inflammatory cytokines like IL-6 and TNF-α, as well as pathways including NF-κB, TLRs, and the NLRP3 inflammasome. Known for their effectiveness in treating mood disorders, TCAs have shown encouraging anti-inflammatory properties. TCAs show great promise in reducing inflammation linked to atherosclerosis through processes that include reducing oxidative stress, suppressing cytokine production, and inhibiting NF-κB activation. The mechanisms underlying the anti-inflammatory effects of TCAs, particularly in relation to atherosclerosis, are complex and multifaceted. TCAs are known to modulate inflammatory pathways through several potential mechanisms. Firstly, they reduce oxidative stress by increasing the activity of antioxidant enzymes and reducing ROS levels, thereby moderating endothelial damage and inflammation. Secondly, TCAs suppress cytokine production, including pro-inflammatory cytokines such as TNF-α, IL-1β, and IL-6, which are key mediators in the pathogenesis of atherosclerosis. This suppression is mediated, at least in part, by their ability to regulate immune cell activation and signaling pathways. Thirdly, TCAs inhibit the activation of NF-κB that regulates the expression of various inflammatory genes. By preventing the translocation of NF-κB to the nucleus, TCAs disrupt the downstream inflammatory cascade, thereby reducing vascular inflammation and plaque formation. Their extended therapeutic possibilities beyond psychiatric diseases are further highlighted by their regulatory effects on TLR signaling and inflammasome activation. However, contradictory results highlight the intricacy of TCAs’ activities and the need for more research, especially with relation to IL-6 levels and adiponectin control. It is crucial that future studies concentrate on the specific molecular processes and therapeutic effects of TCAs in vascular disorders caused by inflammation. In addition to improving cardiovascular health, repurposing TCAs as supplementary therapy for atherosclerosis could also address comorbidities like depression, providing a more comprehensive approach to patient care.

## Figures and Tables

**Table 1:** Summary of IL-6 roles, signaling pathways, and the anti-inflammatory effects of TCAs.

Topic	Mechanisms/Details	Effects	Ref.
Role of IL-6 in Inflammation	Acts as both a pro-inflammatory and anti-inflammatory cytokine, depending on context.	Elevated levels linked to cardiovascular diseases and atherosclerosis.	[34]
	Promotes adhesion molecule expression on endothelial cells.	Facilitates inflammatory cell recruitment to sites of injury.	[6]
	Stimulates production of acute-phase proteins in the liver.	Enhances systemic inflammatory responses.	
IL-6 Signaling Pathways	Classical signaling: IL-6 binds to membrane-bound IL-6R, activates JAK-STAT pathway.	Targeted immune response in specific immune cells.	[35]
	Trans-signaling: IL-6 binds to soluble IL-6R, interacts with gp130 on broader cell types.	Amplifies systemic inflammation and affects diverse cell types.	
Anti-Inflammatory Effects of TCAs	Inhibits NF-κB activation, reducing inflammatory cytokine production.	Attenuates inflammation and improves endothelial function.	[21,23]
	↓ Oxidative stress through various molecular pathways.	Enhances vascular and systemic health.	
TCAs and IL-6 Pathway	Nortriptyline and imipramine have been shown to ↓ IL-6 levels.	Suggests potential anti-inflammatory benefits.	[36]
	Some studies indicate ↑ IL-6 levels with TCAs like duloxetine and amitriptyline.	Highlights variability in response across studies.	[31]
	Imipramine showed no significant impact on IL-6 levels in specific studies.	Demonstrates the complexity of TCA effects on IL-6 pathways.	[37]
Proposed Mechanisms	TCAs inhibit NF-κB, JAK/STAT, MAPK, and PI3K pathways, ↓ IL-6 production.	Exerts anti-inflammatory effects through multiple molecular mechanisms.	[38]

↑: Increase; ↓: Decrease.

**Table 2:** Role of TNF-α in atherosclerosis.

Category	Details	Ref.
Production and Role of TNF-α	Produced by macrophages, lymphocytes, and endothelial cells. Regulates immune responses, cell proliferation, differentiation, and apoptosis. Contributes to atherosclerosis by upregulating VCAM-1, ICAM-1, and E-selectin, promoting leukocyte adhesion and transmigration. Induces IL-1β, IL-6, chemokine, and MMP production.	[21]
NF-κB Activation	TNF-α binds to TNFR1 and TNFR2, activating NF-κB via canonical (IκBα degradation) and non-canonical (NF-κB2/p100-to-p52 processing) pathways. Leads to inflammation, apoptosis, and ROS production. ROS causes endothelial damage and NO inactivation, worsening endothelial dysfunction.	[39,46]
Pathways of TNF-α-Induced Apoptosis	Extrinsic pathway: Mediated by death receptors. Intrinsic pathway: Mitochondrial-mediated apoptosis. Contributes to plaque instability.	
Effects of TCAs on TNF-α	↓ TNF-α levels Inhibit NF-κB activation by stabilizing IκBα, reducing pro-inflammatory gene transcription. Reduce ROS via antioxidant actions (enhancing SOD, catalase). Restore NO bioavailability, improving endothelial function and vasodilation.	[39,41,42]
Clinical Implications	↓ Inflammation and oxidative stress. Stabilization of atherosclerotic plaques. Potential as adjunctive therapy for patients with depression and atherosclerosis.	[21]

**Table 3:** The role of NF-κB in atherosclerosis and the anti-inflammatory effects of TCAs.

**Category**	**Details**	**Ref.**
NF-κB Family and Its Functions	Key transcription factor in inflammation and atherosclerosis. Regulates cytokines, chemokines, adhesion molecules, and apoptosis-related genes. Composed of proteins such as RELA (p65), NF-κB1 (p50; p105), and NF-κB2 (p52; p100).	[54]
Activation Pathways	Canonical Pathway: Triggered by stimuli like TNF-α, interleukins, and LPS. IKK complex phosphorylates IκB, leading to its degradation and NF-κB activation. Non-Canonical Pathway: Stimulated by factors like CD40 ligation and lymphotoxin-β. Relies on p100 processing to p52 via IKK1 and NIK.	[55]
Role in Atherosclerosis	NF-κB activation enhances the transcription of pro-inflammatory genes. Promotes endothelial dysfunction and adhesion molecule expression. Elevated activity was observed in unstable coronary plaques.	[40,41,42,56]
Effects of TCAs on NF-κB	↓ NF-κB activation by inhibiting TNF-α release. Imipramine ↓ the nuclear translocation of p65 and the nuclear/total NF-κB ratio. Tianeptine and amitriptyline suppress the activation of p50, p65, and IκB.	[29,42,52]
Additional Mechanisms of TCAs	Imipramine remodels the IL-10 promoter, inhibiting p50/c-Rel binding. Imipramine blue (IB) reduces NADPH oxidase activity, lowering ROS, a key NF-κB activator. Reduced ROS prevents excessive inflammatory responses.	[57]
Therapeutic Implications	By suppressing NF-κB activation, TCAs mitigate inflammation, oxidative stress, and endothelial dysfunction. Potential benefits extend to conditions like atherosclerosis beyond their antidepressant effects.	[40,41,42]

**Table 5:** Adiponectin in atherosclerosis: functions, therapeutic potential, and the impact of TCAs.

Category	Details	Ref.
Function of Adiponectin	Adipokine is secreted by adipocytes with anti-atherogenic properties. Modulates AMPK and cAMP-PKA pathways to suppress apoptosis, ROS, and NF-κB pathways. Induces NO production, protecting against atherosclerosis.	[72,73]
Therapeutic Potential	Demonstrated by studies to have anti-atherogenic effects. Recognized as a potential therapeutic agent for cardiovascular protection.	[74]
Effects of TCAs on Adiponectin	Doxepin: Shown to suppress adiponectin expression in mice. Imipramine: No significant effect on adiponectin expression observed in vitro.	[74]
Research Gaps	Inconsistent findings regarding TCAs’ influence on adiponectin. Further studies required to clarify the effects of TCAs on adiponectin pathways and their therapeutic implications for atherosclerosis.	[74]

**Table 6:** Role of the NLRP3 inflammasome in inflammation and atherosclerosis.

Factor	Effect	TCA Involvement	Ref.
NLRP3 Activation	Initiates inflammation. Promotes IL-1β and IL-18 release. Triggered by cholesterol crystals.	Amitriptyline and imipramine lower NLRP3 mRNA expression and inflammasome activation. Enhanced autophagy inhibits NLRP3 activation.	[78]
Caspase-1 Activation	Converts pro-IL-1β and pro-IL-18 into active cytokines. Drives inflammatory responses.	Imipramine inhibits caspase-1 activity. Amitriptyline alleviates caspase-1-mediated cytokine release.	[79]
IL-1β and IL-18 Release	Triggers endothelial dysfunction and oxidative stress. Contributes to atherosclerosis.	Imipramine and desipramine lower IL-1β levels. Amitriptyline significantly reduces IL-1β production.	[78]
MMP-1 Production Induction by IL-1β	Exacerbates atherosclerotic plaque instability.	Imipramine prevents IL-1β-induced MMP-1 expression, stabilizing plaques.	[78]
Predictive Role of IL-1β	High baseline IL-1β mRNA levels are associated with poor response to nortriptyline.	Suggested as a predictor of treatment response to TCAs like nortriptyline.	[80]
Conflicting Findings	↑ IL-1 levels were observed in obese mice treated with doxepin.	Results highlight variability in TCAs’ effects.	[74]

**Table 7:** Impact of TCAs on key molecules and pathways in atherosclerosis.

Molecule/ Pathway	Role in Atherosclerosis	Effect of TCAs	Mechanisms of Action of TCAs	Ref.
IL-6	Pro-inflammatory cytokine that ↑ atherosclerosis through various mechanisms, including the induction of adhesion molecule production, chemokine production, and T-cell proliferation	Some studies show that TCAs ↓ IL-6 levels, while others report an ↑ or no change	TCAs may ↓ IL-6 production by inhibiting NF-κB, JAK/STAT, and MAPK/PI3K pathways	[4]
TNF-α	Major pro-inflammatory cytokine that contributes to atherosclerosis by ↑ leukocyte adhesion, macrophage differentiation, and the production of other inflammatory mediators	Most studies indicate that TCAs ↓ TNF-α levels	TCAs inhibit NF-κB activation, scavenge ROS, ↑ antioxidant enzyme activity, and inhibit apoptosis, leading to ↓ inflammation and ↑ endothelial function	[2]
NF-κB	Key transcription factor that regulates the expression of numerous genes involved in inflammation and atherosclerosis, including cytokines, chemokines, and adhesion molecules	TCAs ↓ NF-κB activation	TCAs inhibit TNF-α release, ↓ total NF-κB levels, remodel the IL-10 promoter, inhibit NADPH oxidase activity, and ↓ ROS production	[52]
TLR	Pattern recognition receptors that play a role in inflammation and atherosclerosis by recognizing PAMPs and DAMPs and activating downstream signaling pathways	TCAs inhibit TLR expression, particularly TLR-4 and TLR-2	The exact mechanisms by which TCAs inhibit TLRs are not fully elucidated	[52]
Adiponectin	Adipokine with anti-atherogenic properties due to its effects on AMPK and cAMP-PKA pathways and its ability to suppress apoptosis, ROS production, and NF-κB activation	Doxepin may suppress adiponectin expression, while imipramine may not have an effect	Further research is needed to clarify the impact of TCAs on adiponectin and its related pathways	[52,72]
NLRP3 and IL-1	NLRP3 inflammasome activation leads to the processing and release of pro-inflammatory cytokines IL-1β and IL-18, contributing to endothelial dysfunction and atherosclerosis	TCAs ↓ NLRP3 inflammasome activation, IL-1β, and IL-18 levels	TCAs may inhibit NLRP3 inflammasome activation through autophagy induction, direct inhibition of NLRP3 and IL-1β expression, and prevention of IL-1β-induced MMP-1 production	[79,89]

## Data Availability

The corresponding author will provide the datasets created during and/or analyzed during the current investigation upon reasonable request.

## Abbreviations

The following abbreviations are used in this manuscript:

DAMPs	damage-associated molecular patterns
GABA	γ-Aminobutyric acid
ICAM-1	intercellular adhesion molecule-1
IκBα	inhibitor of nuclear factor kappa B
JAK-STAT	Janus kinase–signal transducer and activator of transcription
MAPK	mitogen-activated protein kinase
MMPs	matrix metalloproteinases
NET	norepinephrine transporter
NIK	NF-κB-inducing kinase
NF-κB	nuclear factor kappa B
NLRP3	NLR family pyrin domain-containing protein 3
NMDA	N-methyl-D-aspartate
NOD	nucleotide-binding oligomerization domain
PAMPs	pathogen-associated molecular patterns
PI3K	phosphoinositide 3-kinase
SERT	serotonin transporter
STAT	signal transducer and activator of transcription
TBK1	TANK-binding kinase-1
TCA	tricyclic antidepressants
TLR	Toll-like receptors
TNFR1	tumor necrosis factor receptor-1
TRADD	receptor-associated death domain protein
VCAM-1	vascular cell adhesion molecule-1
VEGF	vascular endothelial growth factor
